# SMΝΔ7 mice show breathing and airflow defects with significant pathology of respiratory and oral tract tissues

**DOI:** 10.3389/fncel.2026.1844362

**Published:** 2026-06-19

**Authors:** Roxanne Muchow, Michelle Woolridge, Catherine L. Smith, F. Javier Llorente Torres, Dennis Perez-Lopez, Nicole L. Nichols, Monique A. Lorson, Christian L. Lorson

**Affiliations:** 1 Department of Pathobiology and Integrative Biomedical Sciences, College of Veterinary Medicine, University of Missouri, Columbia, MO, United States; 2 Bond Life Sciences Center, University of Missouri, Columbia, MO, United States

**Keywords:** histology, neuromuscular disease, plethysmography, respiration, spinal muscular atrophy

## Abstract

**Introduction:**

Spinal muscular atrophy (SMA) is a neurodegenerative disorder caused by *SMN1* mutations, leading to SMN protein deficiency and motor neuron loss. While progressive weakness, respiratory defects, and oral dysfunction are well-documented in patients, the underlying pathophysiology of breathing and bulbar deficits remains understudied in SMA animal models.

**Methods:**

We evaluated breathing and oral function in the SMN∆7 mouse model of severe SMA. Respiratory parameters and chemoreflexes were assessed via whole-body plethysmography. To identify underlying structural changes, we performed histological analysis on lung tissue, the phrenic and hypoglossal nerves, and the muscles driving respiration and oral function.

**Results:**

SMN∆7 mice exhibited baseline respiratory alterations and chemoreflex deficits. Histological analysis revealed reduced neuromuscular junction (NMJ) occupancy in respiratory and oral muscles, alongside axonal pathology in the phrenic and hypoglossal nerves and structural degradation in lung tissue.

**Discussion:**

These data provide the first physiological and histological evidence of linked respiratory and oral dysfunction in the SMN∆7 mouse. Because these deficits closely approximate the clinical presentation seen in SMA patients, this model represents a valuable tool for testing therapies targeted at bulbar and respiratory failure.

## Graphical abstract

**Figure:**
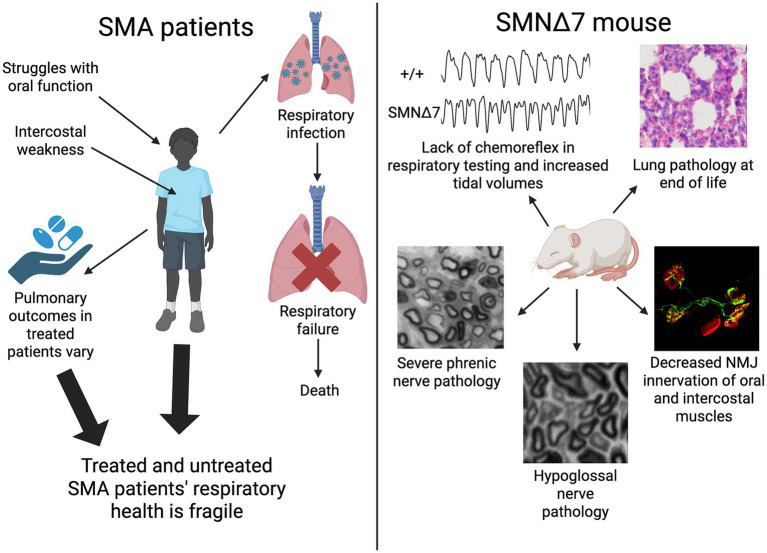

## Introduction

Spinal muscular atrophy (SMA) is a genetic disorder caused by mutations and primarily deletions of the survival motor neuron (*SMN1*) gene, leading to insufficient levels of ubiquitously expressed SMN protein (Crawford and Pardo, 1996; Oskoui et al., 2007; Keinath et al., 2021). The *α*-motor neurons of the ventral horn are highly susceptible to SMN loss, resulting in neuronal degeneration. As SMN is present in all cells, loss of SMN likely affects the homeostasis of many tissues. SMN protein has defined roles in pre-mRNA splicing, translation, mRNA transport, RNA metabolism, and others (Rochette et al., 2001; Kolb et al., 2007; Chaytow et al., 2018). SMA patient clinical symptoms include muscle atrophy, muscle weakness, loss of mobility, weakened head and neck control, altered oral function, and respiratory insufficiency. Respiratory weakness, reduced cough clearance, and swallowing dysfunction are important causes of morbidity and mortality in SMA patients (Wang et al., 2007; Lunn and Wang, 2008; LoMauro et al., 2016; Wijngaarde et al., 2020; Kant-Smits et al., 2026). Despite the prevalence of respiratory and swallowing defects reported in SMA patients, minimal etiological research regarding these deficits has been conducted (McGrattan et al., 2021; Messina et al., 2008; Marti et al., 2024).

Importantly, respiratory and swallowing deficits are commonly reported clinical features in several neuromuscular and neurodegenerative diseases, including spinal muscular atrophy with respiratory distress (SMARD1) and ALS (Kaindl et al., 2008; Porro et al., 2014; Gonzalez de Aguilar et al., 2007; Yunusova et al., 2019; Hermann et al., 2022). While there are similarities in respiratory and swallowing changes reported between these diseases, there are also distinct clinical differences. For instance, SMARD1 and ALS-associated respiratory complications are primarily associated with paralysis of the diaphragm, with weakness of respiratory accessory muscles (intercostals, abdominals) later in the disease (Lechtzin, 2006; Nichols et al., 2013; Pinto and Carvalho, 2014; de Carvalho et al., 2019; Carvalho et al., 1999; Johnson and Mitchell, 2013; Pitt et al., 2003; Kaindl et al., 2008; Perego et al., 2020). In contrast, initial respiratory symptoms in SMA are associated with accessory muscle weakness, followed by diaphragm pathology (McGrattan et al., 2021; Kuru et al., 2009; Schroth, 2009; Darras, 2015). Pre-clinical models of SMARD1 and ALS have shed light on respiratory and swallowing dysfunction and how disease pathology is associated with these alterations, while SMA has received minimal research on this pathology in pre-clinical models (Smith et al., 2022; Ricardez Hernandez et al., 2025; Torres et al., 2025; Keeler et al., 2020; Holbrook et al., 2024; Nichols et al., 2014; Biswas et al., 2024; Tankersley et al., 2007).

There are three FDA-approved therapeutics for SMA, including two modifiers of splicing, Spinraza (nusinersen) and Evrysdi (risdiplam), and Zolgensma/Ivitsma, an AAV9-*SMN* gene therapy (onasemnogene abeparvovec) (Keinath et al., 2021; Schroth et al., 2025; Giess et al., 2024). These therapeutics have greatly improved the duration and quality of life of patients; however, outcomes vary depending on the severity of the disease and timing of treatment. Pulmonary outcomes of treated patients have varied, and the extent of improvement in dysphagia is unclear due to a lack of specification, validation, and sensitivity of measures (Paul et al., 2021; McGrattan et al., 2021; Edel et al., 2025). Inconsistent endpoints for event-free survival across clinical trials hinder our ability to accurately compare outcomes between research groups. Collectively, these studies demonstrate a longitudinal demand for pulmonary support in treated patients, highlighting an important knowledge gap that warrants further preclinical investigation.

In this study, we assessed respiratory pathology in the SMNΔ7 mouse model of SMA (FVB-*SMN2^+/+^ SMNΔ7^+/+^ Smn^−/−^*) (Lee et al., 2011). SMNΔ7 mice have a severe SMA phenotype, with a lifespan of approximately 14 days. SMNΔ7 mice were used in preclinical trials of Spinraza, Evrysdi, and Zolgensma (Passini et al., 2014). Despite extensive research on the SMNΔ7 model, little is known concerning respiratory or swallowing function. Previous studies have shown reduced occupancy of neuromuscular junctions in muscles involved in respiration and oral function, reduced milk sack presence, changes to the phrenic nerve morphology, and decreased diaphragm fiber size (El-Khodor et al., 2008; Woschitz et al., 2022; Ling et al., 2012; Kariya et al., 2008). In this study, we report respiratory deficits in SMNΔ7 mice at post-natal day 7 (P7) and P14 using plethysmography. Our assessment of the diaphragm aligned with previous studies, where the diaphragm neuromuscular junctions (NMJs) are innervated, and diaphragm fiber sizes are reduced. Deficits in plethysmography parameters, peak inspiratory and expiratory flow, led to exploration of the upper respiratory tract and oral cavity. Oral cavity muscles showed a significantly decreased percentage of fully innervated endplates. Additionally, we found significant defects of the phrenic nerve innervating the diaphragm and the hypoglossal nerve innervating the genioglossus (tongue). Collectively, these results provide additional insight into the pathophysiology that gives rise to the respiratory and swallowing defects in this important model of disease.

## Materials and methods

Animal procedures were carried out in accordance with procedures approved by the NIH and MU Animal Care and Use Committee. Animals were housed using standard animal husbandry with free access to water and food. Genotyping of neonatal pups was performed at P0. Genomic DNA was obtained using the DNA isolation protocol from Jackson Labs. All experiments used mice within the time frame of ± 1 day (P7 ± 1 day and P14 ± 1 day).

### SMNΔ7 mice

Heterozygous breeder pairs of mice (*Smn^+/−^; SMN2^+/+^; SMNΔ7^+/+^*), were purchased from JAX® Laboratory (JAX®Stock#005025. FVB. CgGrm7Tg (SMN2)89AhmbSmn1tm1MsdTg(SMN2*delta7) 4299Ahmb/J. Heterozygous breeders were crossed to generate *Smn^−/−^; SMN2^+/+^; SMNΔ7^+/+^* (SMNΔ7) mice. The mouse *Smn* gene was determined using the following primers: FWD 5’-TCT GTG TTC GTG CGT GGT GAC TTT-3′ and REV 5’-CCC ACC ACC TAA GAA AGC CTC AAT-3′. The *SMN2* and *SMNΔ7* transgenes were determined by the presence of the LacZ reporter using the following primers: FWD 5’-CCA ACT TAA TCG CCT TGC AGC ACA-3′ and REV 5′-AAG CGA GTG GCA ACA TGG AAA TCG-3′. Both sets of primers were combined in a standard PCR master mix. 2-step PCR was performed: denaturation for 2 min at 95 °C, 30 s at 95 °C 90 s at 72 °C repeated 35X, then 10 min at 72 °C, and held at 4 °C. Amplimers were separated on 2% agarose gel using gel electrophoresis.

### Plethysmography

Head-out plethysmography was performed at P7 neonatal mice, where all mice were placed in customized head-out pup chambers equipped with warming beds (Data Sciences International/Harvard Bioscience, Holliston, MA) with a low chamber volume for acquiring low-amplitude respiratory signals. Gaps surrounding the neck were sealed using 3 M Impregum F base and catalyst. Head-out plethysmography chambers were selected for P7 mice due to the small size of the animals and the relative lack of sensitivity in the larger chambers for animals of this size. Whole-body plethysmography chambers (Data Sciences International/Harvard Bioscience) were utilized for all mice at P14, as all mice had grown too large to comfortably fit in the head-out chambers. Due to significant size differences at P14, SMNΔ7 mice were tested with an expanded threshold to detect accurate pressure signals, as our typical threshold was too high, resulting in a significant number of missed breathers and lost data across animals, and thus needed to be lowered. Since this information is proprietary to the company, the expanded threshold was utilized based on the manufacturer’s technical department recommendation where the threshold was validated by the manufacturer for both genotypes (Data Sciences International/Harvard Bioscience).

All mice that underwent plethysmography (head-out at P7 and whole-body at P14) were acclimated to the chamber while breathing room air (21% O_2_ + 0% CO_2_ + 79% N_2_; normoxia) for 5 min before ventilatory measurements were recorded for an additional 30 min. The mice were then challenged by exposing them for 5 min to hypercapnia (7% CO_2_, 21% O_2_, balanced N_2_) and subsequently to hypercapnia + hypoxia (7% CO_2_, 10.5% O_2_, balanced N2) for 5 min (gas concentrations controlled by a gas mixer; CWe, Inc., Ardmore, PA). A pressure calibration signal, ambient pressures, and chamber pressures were utilized for automated calculation of breath-by-breath respiratory parameters [frequency (f-breaths/min), inspiratory time (Ti-sec), expiratory time (Te-sec), tidal volume (VT-mL), minute ventilation (V̇E-mL/min), peak inspiratory flow (PIF-mL/s), and peak expiratory flow (PEF-mL-sec)] at 10-s intervals using FinePointe Software (Data Sciences International/Harvard Bioscience, Holliston, MA). VT, V̇E, and mean inspiratory flow (VT/Ti-mL/s) were normalized to body weight (per g). The apnea detection function within FinePointe software was used to identify an additional outcome measure, total number of apneas, that were defined as the absence of at least two inspirations (i.e., a pause in breathing 2x the normalized breath duration threshold) at P14; the apnea detection function within FinePointe cannot be used with the customized chambers used for head-out plethysmography so this outcome measure was not analyzed at P7. The automatically detected apneas were manually reviewed to verify accuracy and exclude rare instances of automated event detection errors, for example, if two shallow breaths were detected as an apnea rather than two individual breaths. Type 0 apneas are defined as a spontaneous apnea (no sigh before it), Type 1 apneas are defined as an apnea that is also a sigh, and Type 2 apneas are defined as a post-sigh apnea. In addition, the apnea detection function within FinePointe software was used to identify the percentage of erratic breathing (defined as any breathing that was not classified as a normal breath, sigh, apnea, or sniff; Data Sciences International/Harvard Bioscience, Holliston, MA) at P14. During normoxia and each challenge, five consecutive time points were selected, averaged, and analyzed. The selection of data points was based on consistency between readings in both frequency and minute ventilation to account for variation in rate and volume (Lind et al., 2020). Data points were selected at the end of exposure periods; normoxia data were selected after 20 min of normoxia, chemoreflex data selection was from the final 2 min of gas exposure. Data were rejected if there was evidence of pressure fluctuations caused by gross body movements (Jacky, 1978; Drorbaugh and Fenn, 1955). Waveforms were generated from raw boxflow data in the FinePointe Software and put into Graphpad Prism.

### Diaphragm fiber area and type immunohistochemistry

An additional set of animals was used for all histological studies, in addition to tissues taken from animals that underwent plethysmography. Animals were anesthetized with isoflurane and sacrificed via cervical dislocation; diaphragms were dissected fresh. Fresh diaphragms were flash frozen in liquid nitrogen, chilled in 2-methylbutane, then embedded in OCT compound (Tissue-Tek© OCT Compound, catalog: 4583, Sakura Finetek USA) and flash frozen again. The antibodies used for muscle fiber type characterization were obtained from the Developmental Studies Hybridoma Bank, created by the NICHD of the NIH and maintained at The University of Iowa, Department of Biology, Iowa City, IA 52242. Tissues were cryosectioned in 10 μm sections and then co-stained with anti-Laminin primary antibody (1:200; catalog: L9393; Millipore Sigma), myosin heavy chain (MyHC) type 1 (1:10, catalog: BA-D5-s, Iowa Developmental Studies Hybridoma Bank), MyHC type 2A (1:50, catalog: SC-71-s, Iowa Developmental Studies Hybridoma Bank), and MyHC type 2B (1:5, catalog: BF-F3-s, Iowa Developmental Studies Hybridoma Bank). Secondary antibody for laminin was Goat anti-Rabbit, Alexa Fluor™ 647 (1:500, catalog: A21244, ThermoFisher Scientific), MyHC type 1 Goat anti-Mouse, Alexa Fluor™ 350 (1:500, catalog: A21140, ThermoFisher Scientific), MyHC type 2A Goat anti-Mouse, Alexa Fluor™ 555 (1:500, catalog: A21127, ThermoFisher Scientific), and MyHC type 2B Goat anti-Mouse, Alexa Fluor™ 488 (1:500, catalog: A21042, ThermoFisher Scientific). For the analysis of embryonic muscle fiber type, an additional fresh 10 μm histological section was co-stained with anti-Laminin primary antibody (1:200; catalog: L9393; Millipore Sigma) and eMyHC (embryonic) (1:5, catalog: F1.652, Iowa Developmental Studies Hybridoma Bank). Secondary antibody for laminin was Goat anti-Rabbit, Alexa Fluor™ 647 (1:500, catalog: A21244, ThermoFisher Scientific) and eMyHC Goat anti-Mouse, Alexa Fluor™ 555 (1:500, catalog: A21127, ThermoFisher Scientific). Images were taken at 10X using a Leica DM5500 B microscope (Leica Microsystem Inc.). Muscle fiber area and diameter were analyzed in a blinded manner using SMASH, semi-automatic muscle analysis using segmentation of histology (Smith and Barton, 2014). SMASH was performed as previously described (Smith and Barton, 2014; Torres et al., 2025). Briefly, segmentation was performed on each image. Fibers were filtered using the following settings for both P7 and P14 time points: minimum fiber area = 100, maximum fiber area = 8,000, eccentricity = 1.1, convexity = 0.7. All fibers outside of these ranges were removed. SMASH identified fiber type for individual fiber by color (blue = type 1; red type = 2A; green type = 2B; for the second slide red = embryonic).

### Neuromuscular junction immunohistochemistry

Animals were anesthetized with isoflurane and sacrificed via cervical dislocation, tissues were dissected, and post-fixed in 4% PFA. Whole-mount preparations were used for NMJ immunohistochemistry. Anti-neurofilament heavy chain (NF-H) (1:2,000, catalog: AB5539, Millipore) and anti-synaptic vesicle 2 (SV2) (1:200, catalog: YE269, Life Technologies) primary antibodies were used. Donkey anti-chicken Alexa Fluor 488 (1:400, catalog: 703-545-155, Jackson ImmunoResearch) and goat anti-rabbit Alexa Fluor 488 (1:200, catalog: 111-545-003, Jackson ImmunoResearch) secondary antibodies were used to label the axon (NF-H) and synaptic terminal (SV2). Acetylcholine receptors were labeled with Alexa Fluor 594-conjugated *α*-bungarotoxin (1:200, catalog: B13423, Life Technologies). Per sample, three individual images were acquired at 40X magnification using a Leica DM5500 B microscope (Leica Microsystem Inc.) and analyzed blindly. Images were analyzed in ImageJ (FIJI) based on the endplate overlap with the synaptic terminal. Endplates with missing overlapping terminals were considered fully denervated (FD), endplates with partial overlap were considered partially innervated (PI), and endplates with complete overlap of signal were considered fully innervated (FI). Representative images were taken on Nikon NSPARC at 40X with 3.8 zoom.

### Phrenic and hypoglossal nerve histology

Animals were anesthetized with isoflurane and sacrificed via cervical dislocation, phrenic, and hypoglossal nerves were fresh dissected prior to branch points on muscles and then fixed with 8% glutaraldehyde in phosphate buffer and embedded in resin (Poly/Bed® 812; catalog:21844-1; Polysciences Inc.). Briefly, nerves were incubated in 2% osmium tetroxide in phosphate buffer for 45 min, followed by rinses in ascending ethanol concentrations (50, 70, 80, 95, and 100%) and propylene oxide. Samples were then incubated in a 1:1 propylene oxide to resin mixture for 1 h, incubated in resin overnight, placed in a resin mold, and cured at 60 °C for 8 h. Resin-embedded nerves were sectioned at 1 μm thickness using the Leica ARTOS 3D ultramicrotome. Semi-thin sections of 1 μm were stained with 0.5X alkaline toluidine blue for 3 min, rinsed with deionized water, cover-slipped with Permount mounting medium (ThermoFisher Scientific), and visualized by light microscopy at 100X magnification (Leica DM5500 B, Leica Microsystems Inc.). Image quantification was performed in a blind manner using the semi-automated MyelTracer software (Kaiser et al., 2021). Myelinated fiber counts were counted within one 100X image per nerve/mouse using ImageJ (FIJI). Between 99–102 axons were quantified per phrenic nerve/mouse, and 200 axons were quantified per hypoglossal nerve/mouse for axon area, G-ratio, myelin thickness, and axon diameter analyses.

### Lung H&E

For lungs, protocols were adapted based on the methods in Karpinski et al. (2014) and Silva et al. (2022). Briefly, animals were anesthetized with isoflurane and sacrificed via cervical dislocation. Fresh non-inflated lung tissues were harvested and post-fixed in 4% PFA for 24 h at 4 °C prior to freezing and embedding. Lungs were progressively dehydrated with consecutive higher ethanol concentrations and then embedded in paraffin. Paraffin-embedded lungs were sectioned at 8 μm. Tissue slides were fixed with acetic acid alcohol for 5 min, rinsed in tap water twice for 15 s, and then hematoxylin (catalog: 411165000, ThermoFisher Scientific) was stained for one minute. Slides were then rinsed in ammonia water for 15 s, dehydrated in 95% ethanol twice for 15 s, and eosin Y (catalog: 152885000, ThermoFisher Scientific) stained for 15 s. Slides were then rinsed twice for 15 s in 95% ethanol, 100% ethanol, and xylene. Tissues were covered with Permount mounting medium (catalog: SP15–500, ThermoFisher Scientific). Three individual images/mouse were acquired at 20X magnification using a Leica DM5500 B microscope (Leica Microsystem Inc.) and analyzed blindly. Quantification of pathology was based on the Silva et al. (2022) protocol. Three 20X images were taken per sample, with the sample meaning one lung section per mouse. Each image was scored 0 (no pathology) to 8 (most pathology) for the following parameters: inflammatory cell presence, hyaline membranes, proteinaceous debris, thickening of the alveolar wall, enhanced injury, hemorrhage, and atelectasis. An inflammatory cell is defined as a visible inflammatory cell in the air and interstitial spaces. Hyaline membrane is defined as an acellular deposit (devoid of hemotoxylin staining) in the alveolar region and stained with eosin. Proteinaceous debris is defined as acellular debris in airspaces. Thickening of the alveolar wall is defined as thickening of the cell layers in the alveolar wall. Enhanced injury is defined as the overall impression of tissue-level injury. Hemorrhage is defined as visible red blood cells in the interstitium or airspaces. Atelectasis is defined as the complete or partial collapse of distal airspace. Severity of pathology was based on the score guide in Silva et al. (2022) with minimum absence of pathology (0–1 score), mild pathology (2–3 score), moderate pathology (4–5 score), pronounced injury (5–6 score), and maximum injury (7–8 score).

### Statistics

All experiments were performed in at least three biological replicates for the reproducibility of data. The statistical analyses performed for each experiment are included within the figure legends using GraphPad Prism. *p* values less than 0.05 were considered statistically significant. The number of animals within cohorts is indicated within the figure legends. The outliers test (ROUT) was performed on all data sets, where all presented data have outliers removed. Outliers were identified and removed from phrenic nerve, hypoglossal nerve, and muscle fiber size and type assessments. Two-way ANOVA with Tukey’s multiple comparisons was performed for plethysmography; we reported multiplicity-adjusted *p* value for each comparison. Erratic breathing and apnea quantification were performed with two-way ANOVA with Sidak’s multiple comparisons. Two-way ANOVA with Sidak’s multiple comparisons was performed for NMJ innervation and muscle fiber type populations (all except embryonic); we reported multiplicity-adjusted *p* value for each comparison. An unpaired, two-tailed t-test, normal Gaussian distribution with Welch’s correction was performed for diaphragm fiber areas and embryonic fiber population, and phrenic and hypoglossal nerve analyses. Unpaired, two-tailed t test normal Gaussian distribution was performed for lung pathology quantification.

## Results

### SMNΔ7 mice showed reduced airflow and lacked a chemoreflex response

**Figure 1:**
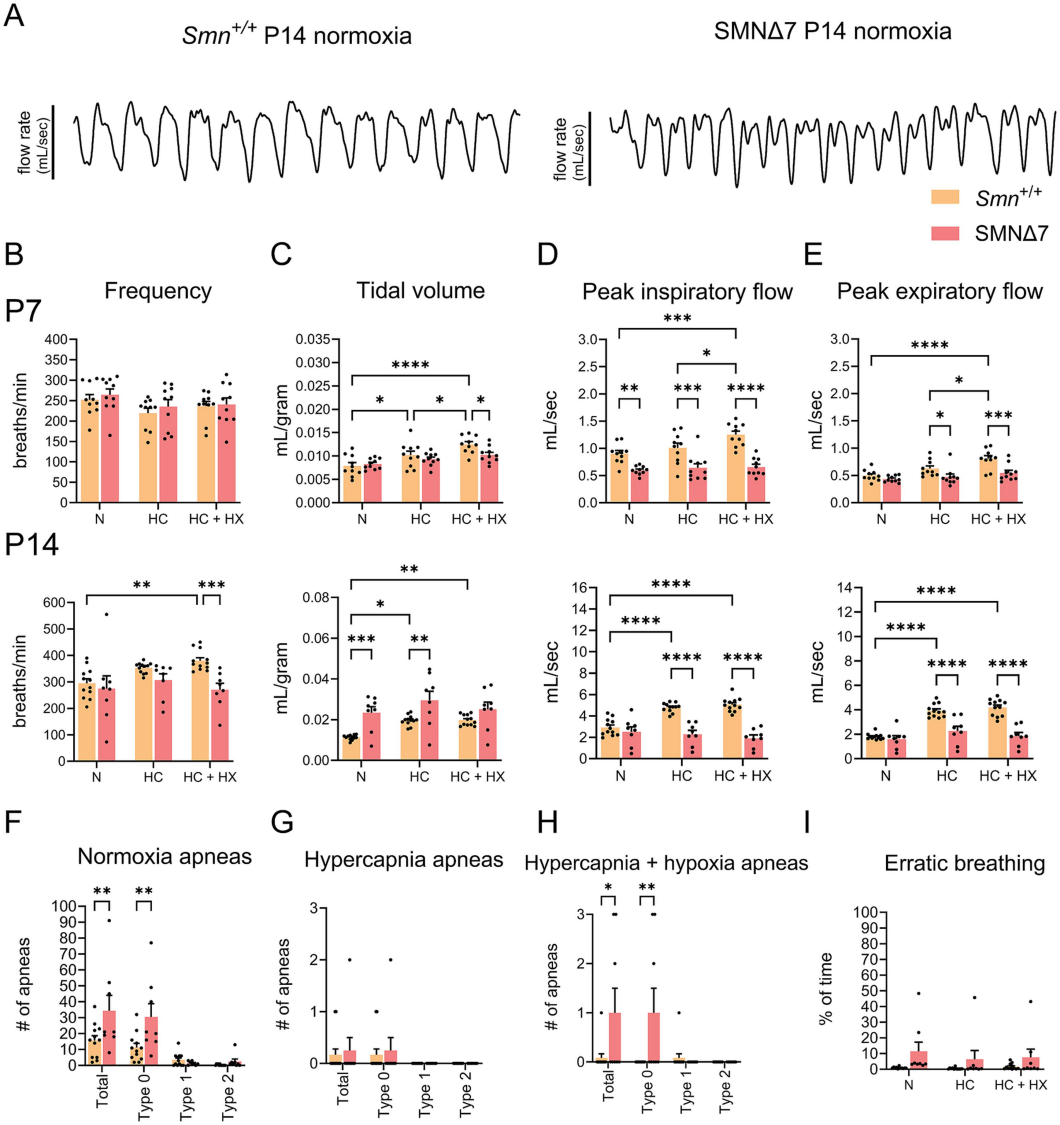
SMNΔ7 mice showed respiratory deficits as quantified by plethysmography. *Smn^+/+^* mice are represented as gold bars, and SMNΔ7 mice are represented as peach bars. **(A)** Waveforms of P7 mice. **(B–E)** P7 plethysmography parameters are located on top (head-out plethysmography), and P14 parameters are located on the bottom (whole-body plethysmography). Respiratory conditions for these studies were N, normoxia; HC, hypercapnia; HC + HX, hypercapnia + hypoxia. **(B)** Frequency measured as breaths per minute. P14 *Smn^+/+^* vs. SMNΔ7 HC + HX *** *p* = 0.0009. P14 *Smn^+/+^* N to HC ** *p* = 0.0097. **(C)** Tidal volume (mL/gram) normalized to weight. P7 *Smn^+/+^* vs. SMNΔ7 HC + HX * *p* = 0.0102. P7 *Smn^+/+^* N to HC * *p* = 0.0240, N to HC + HX **** *p* = <0.0001, HC to HC + HX * *p* = 0.0245. P14 *Smn^+/+^* vs. SMNΔ7 N *** *p* = 0.0003, HC ** *p* = 0.0025. P14 *Smn^+/+^* N to HC * *p* = 0.0104, HC to HC + HX ** *p* = 0.0097. **(D)** Peak inspiratory flow is measured as mL/s. P7 *Smn^+/+^* vs. SMNΔ7 N ** *p* = 0.0014, HC *** *p* = 0.0002, HC + HX **** *p* = <0.0001. P7 *Smn^+/+^* N to HC + HX *** *p* = 0.0009, HC to HC + HX * *p* = 0.0256. P14 *Smn^+/+^* vs. SMNΔ7 HC and HC + HX **** *p* = <0.0001. P14 *Smn^+/+^* N to HC and N to HC + HX **** *p* = <0.0001. **(E)** Peak expiratory flow is measured as mL/s. P7 *Smn^+/+^* vs. SNΔ7 HC * *p* = 0.0184, HC + HX *** *p* = 0.0001. P7 *Smn^+/+^* N to HC + HX **** *p* = <0.0001, HC to HC + HX * *p* = 0.0156. P14 *Smn^+/+^* vs. SMNΔ7 HC and HC + HX **** *p* = <0.0001. P14 *Smn^+/+^* N to HC and N to HC + HX **** *p* = <0.0001. **(F)** Number of apneas during normoxia at P14. Total ** *p =* 0.0068, Type 0 ** *p =* 0.0053. **(G)** Number of apneas during hypercapnia at P14. **(H)** Number of apneas during hypercapnia + hypoxia at P14. Total * *p =* 0.0104, Type 0 ** *p =* 0.0044. **(I)** Percent of time spent in erratic breathing at P14. P7 *n* = 10 for each group. P14 *Smn^+/+^ n* = 12 SMNΔ7 *n* = 8. mL = milliliter, sec = second, min = minute.

We selected the SMNΔ7 mouse model to quantitatively examine SMA respiratory pathophysiology, as this model was commonly used in SMA studies utilizing SMA therapeutics. These mice live approximately 14 days; therefore, a symptomatic time point of 7 days (P7) and an end-of-life time point at day 14 (P14) were chosen for recordings. Due to the small size of mice at P7, head out plethysmography was selected for both wild-type *Smn^+/+^* mice and SMNΔ7 mice, while at P14, mice were too large to be assessed in the pup chambers. Thus, adult chambers were selected for P14 recordings for both wild-type *Smn^+/+^* mice and SMNΔ7 mice. Mice underwent plethysmography, head-out plethysmography at P7 (*n* = 10 per group) and whole-body plethysmography at P14 (*Smn^+/+^ n* = 12 SMNΔ7 *n* = 8), under conditions of normoxia (21% O_2_ + 0% CO_2_ + 79% N_2_) for 30 min, hypercapnia (21% O_2_ + 7% CO_2_, balanced N_2_) for 5 min, and hypercapnia + hypoxia (10.5% O_2_ + 7% CO_2_, balanced N_2_) for 5 min. The following parameters were measured: respiratory frequency, tidal volume, peak inspiratory flow, peak expiratory flow, mean inspiratory flow, minute ventilation, inspiratory time, and expiratory time. Respiratory impairment was more pronounced at P14 than at P7, with functional deficits being significantly greater under hypercapnia and hypercapnia + hypoxia conditions compared to normoxia (Figure 1; Supplementary Figure 1). At P7, all parameters, excluding frequency and inspiratory and expiratory time, showed significant changes between wild-type and SMNΔ7 mice under respiratory challenge conditions (hypercapnia + hypoxia) (Figure 1). These results show that alterations impacting respiration were present, but not yet severe, because respiration was largely preserved under conditions of normoxia in SMNΔ7 mice. At P7, the most significant respiratory changes measured in SMNΔ7 mice were in airflow, with significantly reduced peak inspiratory flow in P7 normoxia and both peak expiratory and inspiratory flow during hypercapnia and hypercapnia + hypoxia testing (Figures 1D,E). Interestingly, P7 mean inspiratory flow was normal in SMNΔ7 mice during normoxia and hypercapnia conditions, suggesting significant upper airway resistance while maintaining continued airflow. P7 SΜΝΔ7 mice did not respond to hypercapnia or hypercapnia + hypoxia conditions for any parameter, demonstrating altered chemoreflex responses (Figure 1; Supplementary Figure 1). At P14, the only significant chemoreflex response was a decrease in expiratory time from normoxia to hypercapnia in SMNΔ7 mice; therefore, SMNΔ7 mice overall lack a chemoreflex across their lifespan (Figure 1; Supplementary Figure 1D). SMNΔ7 mice showed significant respiratory changes, shown by increased minute ventilation, mean inspiratory flow, and tidal volume at P14 during normoxia (Figure 1C; Supplementary Figures 1A,B). Changes in respiratory frequency are also observed at P14 within hypercapnia + hypoxia conditions, while no changes in frequency were noted at P7 during any testing condition (Figures 1A,B). SMNΔ7 mice show prolonged expiratory time at P14 during normoxia, while no change in inspiratory time is shown (Supplementary Figures 1C,D). At P14, SMNΔ7 mice show increases in inspiratory time during hypercapnia and hypercapnia + hypoxia testing (Supplementary Figure 1C).

At P14, there was a significant increase in the number of total apneas and type 0 (spontaneous) apneas during normoxia in SMNΔ7 mice (Figure 1F). During hypercapnia + hypoxia at P14, there was a significant increase in total apneas and type 0 apneas, whereas no significant changes in apneas were observed during hypercapnia alone (Figures 1G,H). There was no significant change found in erratic breathing during any testing condition at P14 (Figure 1I). In summary, SMNΔ7 mice showed respiratory airflow deficits, increased apneas during normoxia and hypercapnia + hypoxia conditions, and a lack of a chemoreflex response that together likely result in unstable breathing conditions, poor oxygenation, and organ stress (Smith et al., 2022; Ricardez Hernandez et al., 2025; Torres et al., 2025).

### Diaphragm muscle fibers showed reduced muscle fiber size

**Figure 2:**
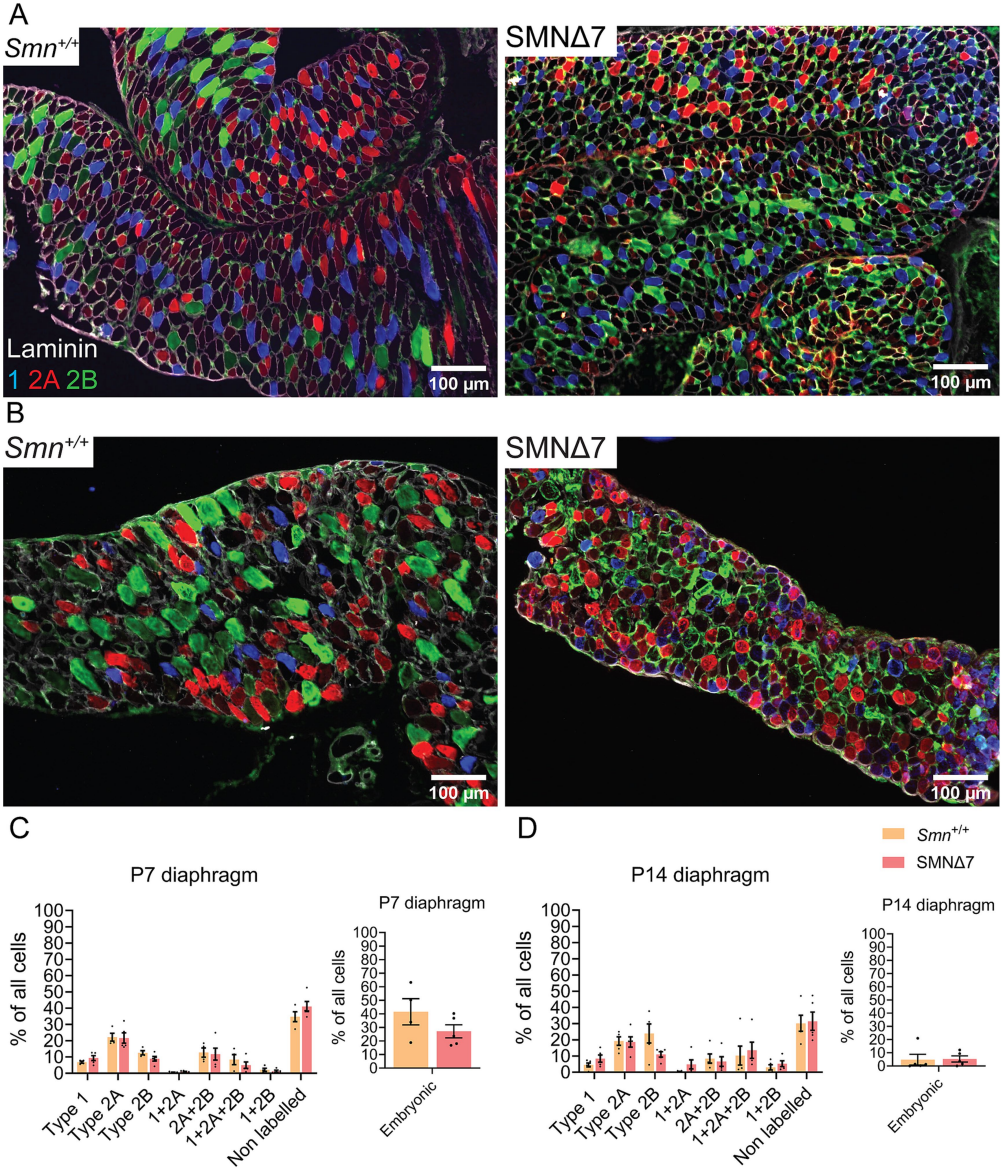
SMNΔ7 mice diaphragm muscle fiber type populations were comparable to wild-type mice. *Smn^+/+^* mice are represented as gold bars, and SMNΔ7 mice are represented as peach bars. **(A)** Representative 10X images of cross sections of P7 diaphragms of *Smn^+/+^* and SMNΔ7 mice. Laminin is represented as white, type I fibers are blue, type 2A fibers are red, and type 2B fibers are green. **(B)** Representative images of the cross-section of P14 diaphragms. **(C)** Diaphragm muscle fiber populations of P7 *Smn^+/+^* and SMNΔ7 mice. No significant changes found in any population. **(D)** Diaphragm muscle fiber populations of P14 *Smn^+/+^* and SMNΔ7 mice. No significant changes found in any population. P7 *Smn^+/+^ n* = 4, SMNΔ7 *n* = 5. P14 *n* = 5 for each group. Quantification of fiber areas found in Table 1.

Previous studies of the SMNΔ7 diaphragm showed reduced muscle fiber size (Passini et al., 2014). We investigated whether specific muscle fiber types were selectively vulnerable to changes or if the overall fiber type composition of the diaphragm was altered. At P7 and P14, changes in muscle fiber type composition were not observed (Figures 2A–D; Supplementary Figure 2). Consistent with previous studies, P7 SMNΔ7 mice (*Smn^+/+^ n* = 4 SMNΔ7 *n* = 5) showed reduced diaphragm muscle fiber size of all muscle fiber types; however, embryonic and type 1 muscle fibers were reduced more than the fast-twitch type 2 fibers (embryonic −38%, type 1–31%, type 2A − 22%, type 2B -24%, and non-labelled −22%) (Table 1). By P14, end-stage for SMNΔ7 mice (*n* = 5 per group), type 1, type 2A, type 2B, and non-labeled diaphragm muscle fibers all showed a two-fold decrease in muscle fiber area (embryonic −41%, type 1–60%, type 2A − 57%, type 2B -65%, and non-labelled −53%) (Table 1). These studies show that initially, embryonic and slow-twitch type 1 diaphragm muscle fibers show the largest reduction in size; however, by end-stage, generalized atrophy across all diaphragm fiber types occurs. This structural decline likely drives the functional impairments captured during plethysmography studies.

**Table 1:** Muscle fiber type of the diaphragm cross sections reported as mean fiber area μm^2^ ± standard deviation.

Fiber type	*Smn^+/+^* P7	SMNΔ7 P7	*Smn^+/+^* P14	SMNΔ7 P14
Total area	486.0 ± 254.4	384.2 ± 188.1 ****	835.6 ± 562.1	376.4 ± 215.6 ****
Embryonic	455.6 ± 184.3	280.9 ± 111.5 ****	682.0 ± 357.0	405.2 ± 241.7 ****
Type 1	522.6 ± 173.6	361.1 ± 122.1 ****	1,047 ± 546.8	421.7 ± 211.5 ****
Type 2A	442.9 ± 179.7	344.0 ± 151.5 ****	857.7 ± 447.3	368.8 ± 225.6 ****
Type 2B	573.6 ± 344.4	433.7 ± 247.0 ****	1,090 ± 728.2	378.6 ± 218.7 ****
Type 1 + 2A	341.4 ± 151.5	271.4 ± 119.2 ns	940.7 ± 297.6	480.0 ± 264.7 *
Type 1 + 2B	405.2 ± 179.5	404.1 ± 173.1 ns	590.5 ± 336.9	384.2 ± 191.2 ***
Type 2A + 2B	318.8 ± 179.3	250.4 ± 117.1 ****	478.8 ± 293.2	249.5 ± 134.8****
Type 1 + 2A + 2B	300.1 ± 150.1	183.4 ± 76.34 ****	377.5 ± 234.5	285.5 ± 141.0 ****
Non-labelled	575.2 ± 260.8	445.9 ± 195.5 ****	878.4 ± 563.3	413.1 ± 237.4 ****

Non-labeled cells are presumed to be a mix of embryonic and type 2X fibers. * *p* = 0.0117, *** *p* = 0.0002 **** *p* < 0.0001. P7 *Smn^+/+^ n* = 4, SMNΔ7 *n* = 5. P14 *n* = 5 for each group.

### NMJ innervation was altered in the oral cavity and the internal intercostal muscles

**Figure 3:**
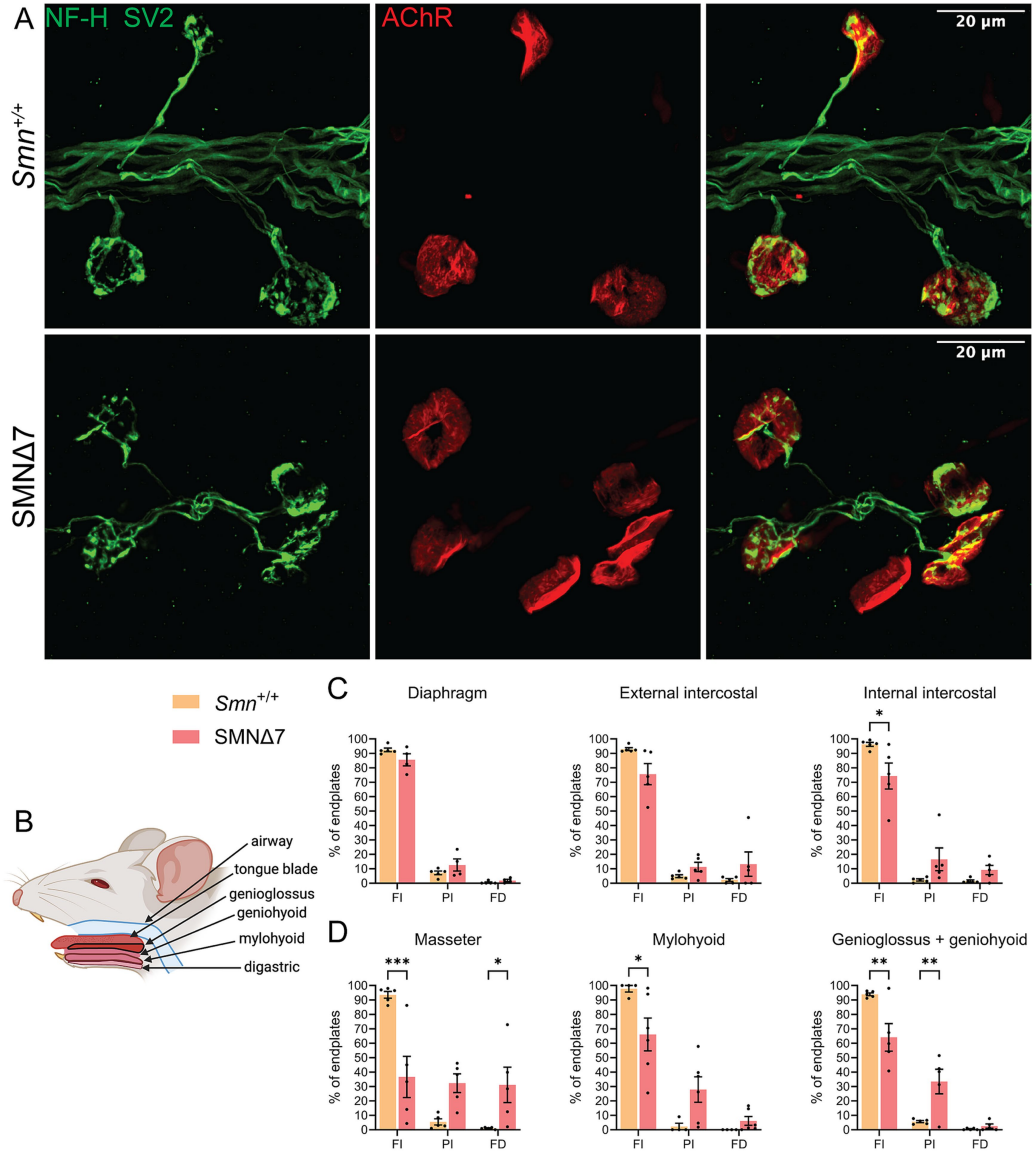
SMNΔ7 oral cavity and internal intercostal muscles showed significant changes in NMJ innervation. *Smn^+/+^* mice are represented as gold bars, and SMNΔ7 mice are represented as peach bars. **(A)** Representative images of P7 mylohyoid NMJs taken at 40X. Green represents neurofilament heavy (NF-H) and synaptic vesicle 2 (SV2), while red represents acetylcholine receptors (AChR). **(C,D)** NMJ quantification as a percentage of analyzed endplates as FI = fully innervated, PI = partially innervated, and FD = fully denervated. **(B)** Cartoon representing oral cavity muscles. **(C)** Quantification of lower respiratory cavity NMJ innervation status. Internal intercostal FI * *p* = 0.0170. **(D)** Quantification of oral cavity and upper respiratory tract NMJ innervation status quantification. Masseter FI *** *p* = 0.0002, FD * *p* = 0.0479. Mylohyoid FI * *p* = 0.0206. Genioglossus+geniohyoid FI ** *p* = 0.0016, PI ** *p* = 0.0032. N = ≥4 for all groups.

The SMNΔ7 respiratory abnormalities measured in plethysmography led us to assess the innervation status of NMJs (Figure 3A). NMJ occupancy was categorized based on the overlap of nerve terminal and muscle endplate signals: a complete overlap identified fully innervated endplates (FI), a partial overlap identified partial innervated endplates (PI), and a lack of overlapping signals identified fully denervated endplates (FD).

Previous studies of SMNΔ7 mice showed that the diaphragm maintained NMJ innervation, while the intercostal muscles showed mild decreases in NMJ innervation (Ling et al., 2012; Kariya et al., 2008). Our analysis aligned with previous findings, showing no significant changes in diaphragm NMJ innervation at P7 (*Smn^+/+^ n* = 5 SMNΔ7 *n* = 4) or P14 (*n* = 5 per group) (Figure 3C; Supplementary Figure 3A). Our studies separated the external intercostal muscles that are activated during inspiration to raise the ribcage from the internal intercostal muscles that are activated during expiration. Interestingly, we observed altered NMJ occupancy only within the internal intercostal muscles of P7 (*n* = 5 per group) and P14 (*n* = 5 per group) SMNΔ7 mice; at P7 74.27% of endplates were fully innervated (*p* = 0.0170) and at P14 83.49% of endplates were fully innervated (*p* = 0.0002) and 14.73% were partially innervated (*p* = <0.0001) (Figure 3C; Supplementary Figure 3A). External intercostals did not show significant changes in NMJ innervation at P7 (*n* = 5 per group) or P14 (*n* = 5 per group) (Figure 3C; Supplementary Figure 3A).

Deficits in peak inspiratory and expiratory flow led us to explore several muscles that contribute to upper respiratory and oral function: the masseter, mylohyoid, genioglossus, and geniohyoid (Figure 3B). The masseter assists with chewing, opening, and closing of the mouth. The masseter showed significant decreases in fully innervated endplates at P7 (*n* = 5 per group) and P14 (*n* = 5 per group) of SMNΔ7 mice; at P7 36.59% of endplates were fully innervated (*p* = 0.0002) and 31.13% of endplates were fully denervated (*p* = 0.0479) and at P14 54.48% of endplates were fully innervated (*p* = <0.0001) and 28.08% of endplates were partially innervated (*p* = 0.0057) (Figure 3D; Supplementary Figure 3B). The mylohyoid, a muscle located underneath the base of the tongue, assists in elevating the hyoid during swallowing. The mylohyoid also showed significant decreases in fully innervated endplates at P7 (*Smn^+/+^ n* = 4 SMNΔ7 *n* = 6) and P14 (*n* = 5 per group) in SMNΔ7 mice; at P7 66.02% of endplates were fully innervated (*p* = 0.0206) and at P14 30.97% of endplates were fully innervated (*p* = <0.0001), 50.42% of endplates were partially innervated (*p* = <0.0001), and 18.60% of endplates were fully denervated (*p* = <0.0001) (Figure 3D; Supplementary Figure 3B). The genioglossus and the geniohyoid make up the base of the tongue and are important for swallowing as well as widening the airway during inspiration. These muscles also showed significant decreases of fully innervated endplates at P7 (*n* = 5 per group) and P14 (*n* = 5 per group) in SMNΔ7 mice; at P7 64.03% of endplates were fully innervated (*p* = 0.0016) and 33.44% of endplates were partially innervated (*p* = 0.0032) and at P14 82.94% of endplates were fully innervated (*p* = 0.0163) and 15.04% of endplates were partially innervated (*p* = 0.0399) (Figure 3D; Supplementary Figure 3B). Taken together, the SMNΔ7 oral muscles evaluated were vulnerable to denervation. The changes in NMJ innervation of these muscles likely contribute to the airflow deficits quantified in plethysmography of SMNΔ7 mice.

### Significant pathology was observed in the phrenic nerve of SMNΔ7 mice

**Figure 4:**
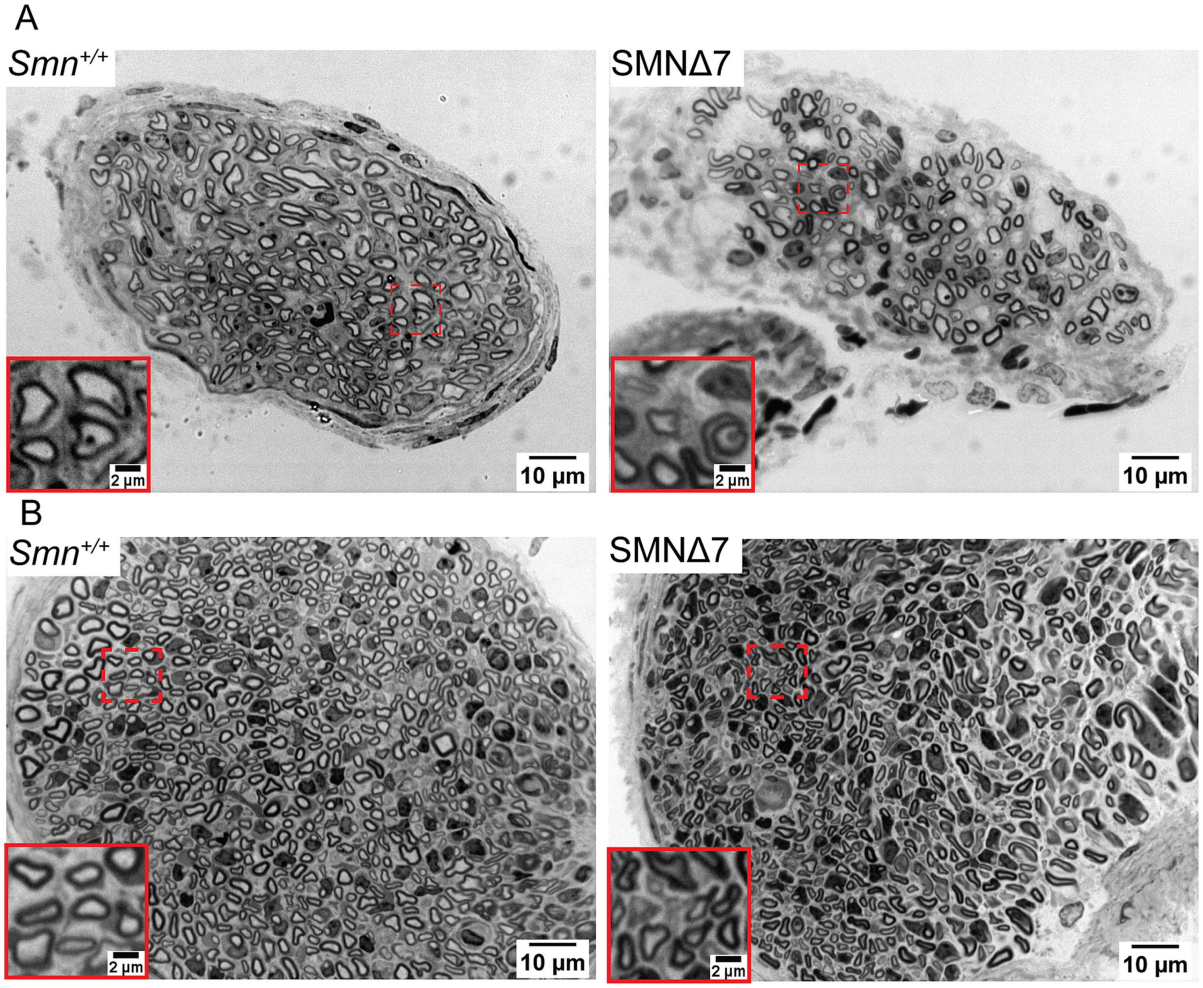
Phrenic and hypoglossal nerves show significant morphological changes. **(A)** Representative images of P7 phrenic nerves. 1 μm thick cross sections stained in toluidine blue and imaged at 100X. **(B)** Representative images of P7 hypoglossal nerves. 1 μm thick cross sections stained in toluidine blue and imaged at 100X. Quantification of nerves is found in Table 2 (phrenic) and 3 (hypoglossal). Phrenic nerve P7 *Smn^+/+^ n* = 4 SMNΔ7 *n* = 6, P14 *n* = 5 per group. Hypoglossal nerve P7 *Smn^+/+^ n* = 4 SMNΔ7 *n* = 5, P14 *Smn^+/+^ n* = 4 SMNΔ7 *n* = 5.

The SMNΔ7 diaphragm muscle fibers were significantly reduced in size while the neuromuscular junctions maintained diaphragm innervation. To determine whether there was phrenic nerve pathology, we assessed phrenic nerve axon area, diameter, G-ratio, myelin thickness, and the number of myelinated fibers/mouse cross-section. Previous reports showed SMNΔ7 P0 phrenic nerve axon populations were not reduced; however, by P14, the axonal population was significantly decreased (Kariya et al., 2008). Our studies examined the phrenic nerve at P7 (*Smn^+/+^ n* = 4 SMNΔ7 *n* = 6) and P14 (*n* = 5 per group) (Figure 4A; Supplementary Figure 4A). SMNΔ7 P7 phrenic nerves showed reduced axon area (−39%), diameter (−23%), G-ratio (−7%), myelin thickness (−7%), and the number of myelinated fibers/mouse cross-section (−23%) (Figure 4A; Table 2). At P14, there was a reduction of area (−59%), diameter (−36%), myelin thickness (−33%), and the number of myelinated fibers/mouse cross-section (−25%). Axon area and myelin thickness showed the most dramatic changes at P14 (Table 2). The significant reduction in axon size, in the number of myelinated axons, and decreased myelin thickness of the phrenic nerve in SMNΔ7 mice suggests that signal transmission from the respiratory center in the brain to phrenic motor neurons and ultimately to the diaphragm would be compromised.

**Table 2:** Phrenic nerve cross sections reported as mean values ± standard deviation.

Age	*Smn^+/+^*	SMNΔ7
Axon area (μm^2^)		
P7	2.542 ± 1.138	1.560 ± 0.9665 ****
P14	4.342 ± 2.060	1.783 ± 0.7795 ****
Axon diameter (μm)		
P7	1.754 ± 0.3993	1.347 ± 0.4147 ****
P14	2.286 ± 0.5498	1.472 ± 0.3202 ****
G-ratio		
P7	0.5673 ± 0.06663	0.5256 ± 0.09063 ****
P14	0.5169 ± 0.08068	0.5136 ± 0.08507 ns
Myelin thickness (μm)		
P7	1.438 ± 0.2421	1.333 ± 0.3359 ****
P14	2.128 ± 0.4728	1.428 ± 0.3296 ****
Myelinated axon count		
P7	196.8 ± 16.13	152.3 ± 28.70 *
P14	202.6 ± 31.71	152.0 ± 23.69 *

Myelinated axon count is one 100X image per nerve/mouse quantified. P7 myelinated axon count * *p* = 0.0144, P14 myelinated axon count *p = 0.0230, **** *p* < 0.0001, ns = no significance. P7 *Smn^+/+^ n* = 4, SMNΔ7 *n* = 6. P14 *n* = 5 for each group.

### The hypoglossal nerve showed significant morphological alterations

Decreases in fully innervated endplates in the genioglossus and geniohyoid led us to investigate the hypoglossal nerve that innervates both muscles. While there were significant changes in hypoglossal axon area, diameter, and G-ratio in P7 (*Smn^+/+^ n* = 4 SMNΔ7 *n* = 5) and P14 (*Smn^+/+^ n* = 4 SMNΔ7 *n* = 5) SMNΔ7 mice, the number of myelinated axons/cross-section was not significantly different between wild-type and SMNΔ7 mice (Figure 4B; Supplementary Figure 4B; Table 3). At P7, myelin thickness was comparable to wild-type; by P14, there was a significant increase in myelin thickness (Table 3). Axon area was most significantly affected (−27%) compared to axon diameter (−15% at P7, −16% at P14) and G-ratio (−10% at P7, −8% at P14). These results suggest that changes in axonal morphology, not myelin, caused the decreased G-ratio in SMNΔ7 mice. Morphological alterations in the hypoglossal nerve are likely contributing to altered NMJ innervation and muscle function in the genioglossus and geniohyoid. These results also support that, alongside varying NMJ pathology per muscle, peripheral nerves do not show equivalent pathology.

**Table 3:** Hypoglossal nerve cross sections are reported as mean values ± standard deviation.

Age	*Smn^+/+^*	SMNΔ7
Axon area (μm^2^)		
P7	1.164 ± 0.6269	0.8431 ± 0.5472 ****
P14	2.825 ± 1.461	2.041 ± 1.065 ****
Axon diameter (μm)		
P7	1.173 ± 0.3274	0.9801 ± 0.3359 ****
P14	1.834 ± 0.4838	1.557 ± 0.4186 ****
G-ratio		
P7	0.4402 ± 0.09888	0.3960 ± 0.1137 ****
P14	0.5310 ± 0.08432	0.4903 ± 0.1153 ****
Myelin thickness (μm)		
P7	1.524 ± 0.4231	1.544 ± 0.4278 ns
P14	1.620 ± 0.4149	1.673 ± 0.5475 *
Myelinated axon count		
P7	551.5 ± 215.7	514.6 ± 201.6 ns
P14	678.0 ± 133.5	676.0 ± 222.7 ns

Myelinated axon count is one 100X image per nerve/mouse quantified. * *p* = 0.0216, **** *p* < 0.0001, ns = no significance. P7 and P14 *Smn^+/+^ n* = 4, SMNΔ7 *n* = 5.

### The lungs showed disease pathology in SMNΔ7 mice

**Figure 5:**
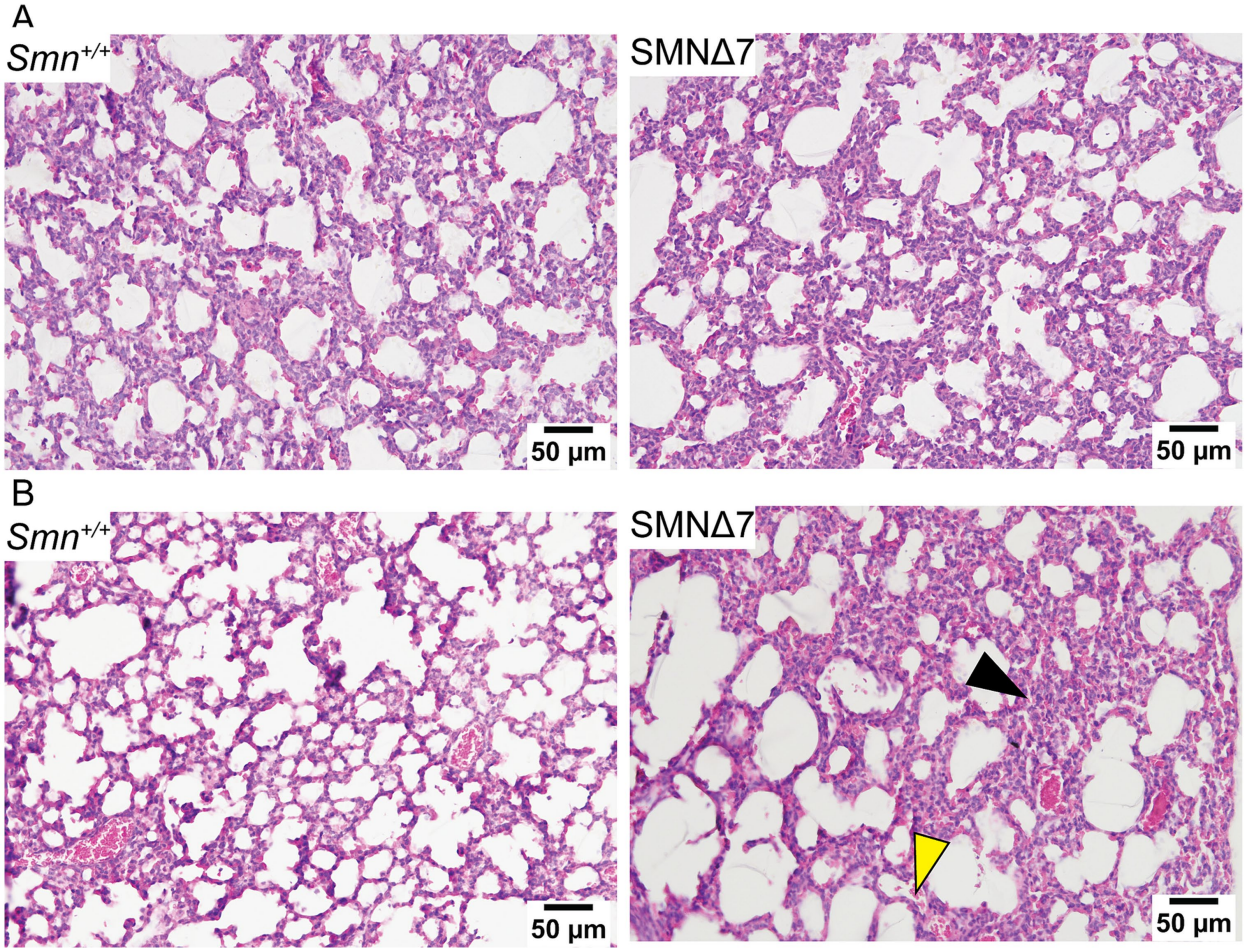
SMNΔ7 lung H&E shows morphological changes at P14. **(A,B)** Representative images of lungs that were paraffin-embedded and H&E-stained. **(A)** P7 lungs. **(B)** P14 lungs. The black arrow points to the collapse of airspaces (atelectasis), and the yellow arrow points to hemorrhage. Quantification of lung tissues is found in Table 4; P7 *n* = 4 per group, P14 *n* = 5 per group.

SMA patients are vulnerable to respiratory infections due to their struggles with cough clearance, aspirations, dysphagia, and recurrent infections, which can exacerbate muscle weakness (Wang et al., 2007; Wijngaarde et al., 2020; Kant-Smits et al., 2026). Furthermore, respiratory problems are a major contributor to morbidity and mortality in SMA patients. To assess whether lung tissue showed pathological changes in SMNΔ7 mice, we examined P7 (*n* = 4 per group) and P14 (*n* = 5 per group) lungs using H&E staining to examine inflammatory cells, thickening of the alveolar wall (cell layer thickness), enhanced injury (tissue injury), proteinaceous debris, presence of a hyaline membrane (acellular deposits in alveolar region), hemorrhage and atelectasis (complete or partial collapse of air spaces) (Figure 5; Table 4). At P7, there were largely no pathological changes found in SMNΔ7 mice; however, by P14, the total pathological score was significantly increased. At P14, hemorrhage and enhanced injury showed significant increases in SMNΔ7 mice, while inflammatory cells, hyaline membranes, proteinaceous debris, thickening of the alveolar wall, and atelectasis did not show significant differences (Figure 5B; Table 4). This evidence demonstrates that respiratory defects shown in plethysmography do not lead to significant lung pathology until the end of life for SMNΔ7 mice.

**Table 4:** Lung H&E pathology reported as mean values with ± standard deviation.

Tissue type	*Smn^+/+^* P7	SMNΔ7 P7	*Smn^+/+^* P14	SMNΔ7 P14
Total score	10.83 ± 4.694	11.5 ± 3.892 ns	7.467 ± 1.464	13.60 ± 3.122 **
Inflammatory cell	1.167 ± 0.7935	1.0 ± 0.2722 ns	0.7333 ± 0.1491	1.2 ± 0.5578 ns
Hyaline membranes	0.4167 ± 0.50	1.0 ± 0.3849 ns	0.2667 ± 0.2789	0.8667 ± 0.6055 ns
Proteinaceous debris	1.5 ± 0.4303	1.25 ± 0.3191 ns	1.267 ± 0.4346	1.867 ± 0.6055 ns
Thickening of alveolar wall	2.5 ± 1.036	2.75 ± 0.9954 ns	1.4 ± 0.2789	2.333 ± 1.155 ns
Enhanced injury	2.417 ± 1.708	2.667 ± 0.9027 ns	1.6 ± 0.4346	2.8 ± 0.9603 *
Hemorrhage	2.0 ± 2.018	1.583 ± 1.198 ns	1.733 ± 0.6412	3.467 ± 1.325 *
Atelectasis	0.8333 ± 1.0	1.25 ± 1.287 ns	0.4467 ± 0.3801	1.067 ± 0.7601 ns

P14 total score ** *p* = 0.0041, P14 enhanced injury * *p* = 0.0344, P14 hemorrhage * *p* = 0.030. P7 *n* = 4 for each group, P14 *n* = 5 for each group. Three 20X images were quantified, with each parameter scored 0 through 8. Total score is reported as the average of the three images (minimum score 0 to maximum score of 56). Parameters are reported as the total score of all three images (minimum score 0 to maximum score of 8). NS, not significant.

## Discussion

Our plethysmography studies showed respiratory deficiencies at P7 and P14 in the SMNΔ7 mouse model for the first time. Histological assessment of tissues of the respiratory and oral tract showed significant pathology with differences observed between tissues. NMJ denervation of the diaphragm was not present, while decreased diaphragm muscle size and severe phrenic nerve axon pathology likely contributed toward respiratory changes in SMNΔ7 mice. The genioglossus and geniohyoid muscles showed significant NMJ denervation with hypoglossal nerve axon pathology, suggesting oral function and/or airflow changes are present in SMNΔ7 mice.

Separate cohorts of mice were used for plethysmography studies and histological assessments due to the fragility of neonatal tissues. This limits our capability to have direct structure-to-function correlations within our study. Future studies including a cohort of animals that receive *in vivo* functional assays and post-mortem histological assessments is ideal. Interestingly, our studies showed an apparent mismatch of severe histological alterations in multiple peripheral respiratory tissues, while plethysmography findings did not show a severe phenotype. This structure-to-function dissonance may be suggestive of compensatory plasticity, possibly occurring within central respiratory networks, which should be explored in future studies.

Previously, no changes in respiratory rate were seen in SMNΔ7 mice based on abdominal and thoracic movement (El-Khodor et al., 2008). Whole-body plethysmography performed in N11/N46 SMA mice found a response to hypoxia at both P1 and P7, whereas our testing of SMNΔ7 mice at P7 via head-out plethysmography and P14 via whole-body plethysmography did not show a significant chemoreflex response during hypercapnia alone or during hypercapnia + hypoxia (Michaud et al., 2010). While other plethysmography recordings did not show chemoreflex, it is possible that chemoreflex response may be present in other assessments, such as electrophysiological recordings of respiratory circuitry. Plethysmography recordings alone cannot distinguish between deficits in chemosensory processing, central integration, or downstream motor input. Additionally, N11/N46 SMA mice showed decreased minute ventilation while SMNΔ7 mice showed increased minute ventilation at P14. N11/N46 SMA mice showed increased apnea duration at P7 during normoxia; similarly, SMNΔ7 at P14 in our study showed an increased number of apneas during normoxia. Our studies focused on peripheral contributions to respiratory phenotype, while central respiratory networks are likely playing a role in our findings. Evidence of apneas and impaired chemoreflex responses implicates involvement of central circuitry. Assessments of central respiratory networks have remained unexplored in the SMNΔ7 mice and are an important consideration for future studies. Though changes to brain size, morphology, neural activity, glial activation, and protein expression have been seen in the brains of various mouse models of SMA previously (Wishart et al., 2010; Tharaneetharan et al., 2021; d'Errico et al., 2013). Furthermore, coculture of SMNΔ7 mice astrocytes and neurons has shown alterations in the GABAergic inhibition system (Menduti et al., 2026).

Despite N11/N46 mice having a similar average lifespan of 14 days, the N11/N46 mice are less severely affected in neuromuscular defects compared to SMNΔ7 mice, as N11/N46 mice showed no deficits in righting reflex and less lumbar motor neuron loss (Michaud et al., 2010). SMNΔ7 and N11/N46 mice are of different congenic backgrounds, FVB/N and C57BL/6 N, respectively, which could also be contributing to differing phenotypes. Altered phenotypes were found in comparison of *Smn*^2B/−^ mice of FVB vs. C57BL/6 background with the FVB *Smn*^2B/−^ mice showing a more severe phenotype than C57BL/6 *Smn*^2B/−^ mice (Eshraghi et al., 2016).

We compared plethysmography recordings and tissue pathology between age-matched SMNΔ7 and FVB-*Ighmbp2*^nmd/nmd^ (FVB-*nmd*) models (Muchow et al. unpublished 2026). The FVB-*nmd* is a severe model of spinal muscular atrophy with respiratory distress type 1 (SMARD1), a disease caused by mutations in the *Ighmbp2* gene (Cox et al., 1998; Shababi et al., 2019). Comparison of the SMNΔ7 mouse with reduced SMN protein and the FVB-*nmd* model with reduced IGHMBP2 protein offers an interesting perspective on the impact of SMN vs. IGHMBP2 loss on the respiratory system. Both models are a severe phenotypes, with FVB-*nmd* mice living around 19 days, though neither model’s respiratory function has had sufficient investigation. Interestingly, SMNΔ7 mice showed more severe tissue pathology and less severe respiratory deficits in plethysmography, with the opposite observed for FVB-*nmd* mice. The most surprising difference was observed in the phrenic nerve between SMNΔ7 and FVB-*nmd* mice. The gross appearance of the SMNΔ7 phrenic nerve cross sections shows remarkable differences compared to the wild type. Considering the severe breathing deficits of the FVB-*nmd*, we would’ve expected their nerves to look more similar to the SMNΔ7 model’s. It remains unclear why the SMNΔ7 mice show severe histological changes of the phrenic nerve despite having only mild respiratory defects. Previously, SMNΔ7 E18.5 embryos showed normal phrenic nerve innervation of the diaphragm; by P0, around 10 % of diaphragm NMJs showed pre-synaptic defects (Kariya et al., 2008). The phrenic nerve demonstrated a reduced number of axons and loss of large-caliber axons at P14 in these mice (Kariya et al., 2008). Interestingly, *Ighmbp2^R604X/R604X^* SMARD1 mice do not show decreases in phrenic nerve axonal populations (Torres et al., 2025). SOD1^G93A^ ALS rats show decreases in phrenic nerve myelinated fiber population at the end of life, and both *Optn^−/−^* and TDP-43^A315T^ ALS mice showed decreased G-ratio in phrenic and hypoglossal nerves (Llado et al., 2006; Biswas et al., 2024). Similar to *Optn^−/−^* and TDP-43^A315T^ ALS mice, our study of SMNΔ7 mice showed decreased G-ratio at P7 with significant decreases in axon area and diameter, likely contributing to decreased NMJ innervation seen in the geniohyoid and genioglossus. Altogether, multiple neurodegenerative disease models show pathology of the phrenic or hypoglossal nerve, though each model appears to have distinct pathology and respiratory phenotypes. While comparison of multiple disease models is informative, these models differ in their underlying molecular mechanisms, which likely explains much of the phenotypic variability observed.

The SMNΔ7 model lifespan averages around 14 days, while the FVB-*nmd* model lives several days longer, about 19–20 days. The answers to why the SMNΔ7 model dies so soon in reference to their mild respiratory deficits and how the FVB-*nmd* model has severe respiratory defects and is able to live longer are unknown. Notably, SMNΔ7 mice have shown decreased milk sac presence from P6 to the end of life, indicating nutritional deficiency, which likely contributes to early death (El-Khodor et al., 2008). While respiratory deficits are likely playing a role in the death of SMNΔ7 mice, the cause of death is likely multifactorial, involving several organ systems. While SMNΔ7 mice have been used in preclinical studies for all FDA-approved SMA therapeutics, their severe phenotype does not fully capture the diverse clinical presentations seen in patients (Passini et al., 2014). Evaluating respiratory function across multiple SMA models with varying neuromuscular deficits and lifespans would provide a more comprehensive understanding of the disease severity and clinical spectrum observed in human populations.

SMA causes the degeneration of motor neurons, resulting in downstream muscular atrophy and weakness. SMNΔ7 mice, in addition to the Taiwanese SMA model, have shown reduced soma sizes and reduced populations of motor neurons in the cervical spinal cord, where the phrenic motor nucleus is located (Baumer et al., 2009; d'Errico et al., 2013; Powis and Gillingwater, 2016). Both our study and Kariya et al. showed decreased phrenic nerve axonal populations in SMNΔ7 mice, demonstrating the downstream pathology from the loss of cervical motor neurons in the spinal cord, suggesting that the phrenic motor nucleus is affected by disease (Kariya et al., 2008). In our study, several tissues found downstream of the spinal cord and brainstem, including phrenic and hypoglossal nerves, external intercostals, and multiple oral cavity muscles, showed significant pathology. It remains unclear why the diaphragm NMJs are preserved; the diaphragm shows decreased fiber areas in combination with phrenic nerve pathology, which is likely contributing to respiratory defects observed with plethysmography. However, because our study only assessed NMJ innervation status, other aspects of NMJ morphology, such as fragmentation, endplate size, and pre- to post-synaptic alignment, may also be altered and could consequently influence the observed respiratory phenotype. Within the SMNΔ7 mouse model in our study, there were mice that appeared to be in worse health on visual assessment, and the severity of tissue pathology varied. Vulnerability of tissues and the extent or type of pathology vary between mouse models as well as within SMA patient populations (Woschitz et al., 2022; Lee et al., 2025). H&E of the SMA type 1 patient diaphragm showed overall preservation with only mild decreases in myofiber diameter (Lee et al., 2025). Simultaneously, another SMA type 1 patient who was maintained on respiratory support for 17 years showed overall diaphragm preservation in H&E and preservation of type 2 fibers, though multiple type 1 fibers were described as putative target or targetoid, suggestive of early denervation. Lee et al. (2025) also found that in an SMA type 1 patient’s intercostals, there was a dramatic increase in the conversion to type 1 fibers and a lack of type 2 fibers. While the diaphragm is considered to be largely spared in patients, alterations in diaphragm neuromuscular junction (NMJ) innervation and diaphragm muscle fiber atrophy were observed in several SMA mouse models (Kariya et al., 2008; McGovern et al., 2008; Voigt et al., 2010; Michaud et al., 2010; Neve et al., 2016).

Despite the prevalence of respiratory and swallowing defects seen in patients of both SMA and SMARD1, minimal etiological research regarding these deficits has been performed. In terms of clinical presentation of respiratory defects, SMA patients have shown increased tidal volumes from their bell-shaped chests due to intercostal weakness, as well as increased aspirations leading to respiratory infections and ultimately respiratory failure and death (McGrattan et al., 2021). In addition, SMA patients demonstrate both obstructive and central apneas (Gurbani et al., 2021; Guo et al., 2023; Abati et al., 2024; Trucco et al., 2024). Though Trucco et al. (2024) notes that “the respiratory pattern of SMA patients during sleep is characterized by events that are not defined by the American Association of Sleep Medicine (AASM) and thus not standardized, they are the expression of the peculiar muscle involvement in SMA”. Our study showed an increased number of apneas, specifically of type 0 spontaneous apneas, during normoxia in P14 SMNΔ7 mice, which is consistent with what is observed in SMA patients. Furthermore, sleep management related to respiratory defects is an important clinical consideration for SMA patients. Respiratory muscle weakness predisposes patients to sleep-disordered breathing. However, explicit alterations in sleep architecture, including reduced REM sleep, increased N1 stage, prolonged sleep latency, and abnormal cyclic alternating pattern (CAP) indices, suggest that central nervous system dysfunction also contributes to sleep impairment in SMA (Gnazzo et al., 2026; Verrillo et al., 2016).

In addition to respiratory defects and sleep disordered breathing, SMA patients may struggle with oral function and swallowing. Videofluoroscopic swallowing studies in SMA patients have shown swallowing deficits, including: aspiration, poor bolus formation, impaired laryngeal elevation, and delayed pharyngeal phase (Choi et al., 2020; McGrattan et al., 2021). Furthermore, SMA patients have shown difficulty with opening the mouth, choking, difficulty chewing, and a need for dietary modifications (Messina et al., 2008; Marti et al., 2024). Analysis of a type 1 SMA patient tissue found overall diaphragm preservation, while intercostals showed moderate pathology, including increased fatty-infiltrates and denervation atrophy (Lee et al., 2025). This study also found major axonal loss in the hypoglossal nerve of a type 1 SMA patient, mild pathology of the mylohyoid with many other ventral neck and infrahyoid muscles spared. Our studies of SMNΔ7 mice recapitulate the overall preservation of the diaphragm with vulnerability in the mylohyoid and intercostals, though the hypoglossal nerve of the SMNΔ7 mice did not have as severe pathology as shown in the type 1 SMA patient. Previously, the masseter and intercostal muscles showed decreased fully innervated NMJs in the SMNΔ7 model and the *Smn^2B/−^* models (Kariya et al., 2008; Woschitz et al., 2022) while SMNΔ7 day 5, day 10, and day 14 mice did not show significant NMJ denervation of tongue muscles (Comley et al., 2016). There are substantial records of clinical symptoms related to respiratory and oral dysfunction in SMA patients. Yet the scientific investigation of these issues is minimal in SMA, while models of several other diseases have already explored respiratory or oral function.

As of 2026, 3 approved treatments exist for SMA patients. Importantly, the timing of treatment and subtype or severity of SMA both play a role in the extent of improvement in treatment, with early treatment and less severe SMA types having better outcomes. Treatment studies have shown improvement in lifespan and muscle strength, among other benefits, but pulmonary outcomes vary (Paul et al., 2021; McGrattan et al., 2021; Giess et al., 2024; Lagae et al., 2024; Edel et al., 2025). Studies of patients using nusinersen or risdiplam have demonstrated that there will be some patients who will require increased pulmonary and nutritional support over their lives (Paul et al., 2021, McGrattan et al., 2021, Giess et al., 2024, Lagae et al., 2024, Edel et al., 2025). A systematic review of sleep phenotypes and respiratory disturbances in treated SMA patients found improvement in some sleep parameters, such as improved sleep oxygenation, though residual sleep disordered breathing remained (Gnazzo et al., 2026). These studies suggest that while disease-modifying therapies may improve or stabilize motor function, respiratory and sleep health may require separate or additional intervention. Oral function and swallowing appear to improve in pre-symptomatically treated patients, while post-symptomatically treated patients show varying results (Marti et al., 2026). Though conclusions were difficult to determine as bulbar outcomes were not recorded in many studies, and the assessment types varied across studies. Furthermore, nusinersen was the first FDA-approved treatment in 2016, long-term outcomes of treated patients are evolving, and new phenotypes of treated patients have emerged (Tizzano and Finkel, 2017). SMA patients treated with AAV9-*SMN* (Zolgensma), an FDA-approved gene therapy treatment, have shown improved swallowing behaviors. There are limited studies in this population, and published studies lack a clear definition of measured dysphagia and lack baseline comparisons (McGrattan et al., 2021; Messina et al., 2008; Marti et al., 2024). Regardless of the improvement with treatments, treated SMA patients are still medically fragile and vulnerable. Continued investigation of respiratory and oral function in patients and models is necessary to continue to improve the quality of life of patients.

Our experiments have demonstrated deficits in respiration as well as respiratory and oral tissue pathology in the SMNΔ7 mouse model. Due to limited preclinical research focus on respiratory and oral dysfunction, there are many unanswered questions left. For one, what molecular pathways are implicated in respiratory dysfunction remains unclear. Furthermore, it is unclear whether lung tissue is impacted by the reduction of SMN or if the lung pathology noted was due to respiratory defects, reduced cough clearance, and aspirations. Future studies need to provide further assessment of swallowing and nutritional defects, gaps in treated models, and molecular pathways.

## Data Availability

The raw data supporting the conclusions of this article will be made available by the authors, without undue reservation.
