# Supplementary material for: SMΝΔ7 mice show breathing and airflow defects with significant pathology of respiratory and oral tract tissues
Source: Front Cell Neurosci. 2026 Jun 19;20:1844362. doi: 10.3389/fncel.2026.1844362 (PMC13328262; doi:10.3389/fncel.2026.1844362)
Supplement: Supplementary file 1 [file Supplementary_file_1.docx]

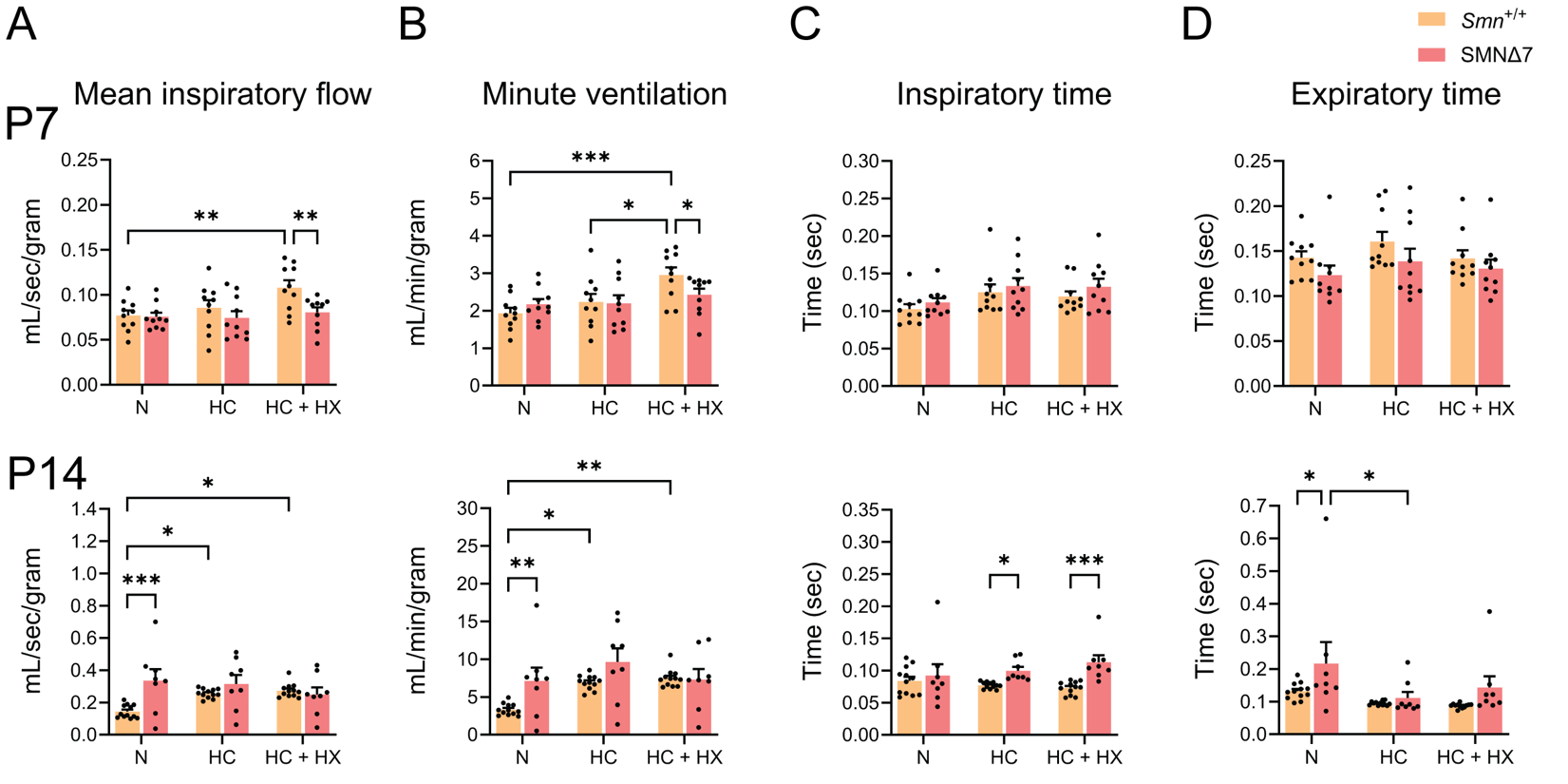


**Supplemental 1** Plethysmography parameters measured at P7 and P14. *Smn^+/+^* mice are represented as gold bars and SMNΔ7 mice are represented as peach bars. Respiratory conditions for these studies were N = normoxia, HC = hypercapnia, HC+HX = hypercapnia + hypoxia (**A**) Mean inspiratory flow (mL/sec/gram) normalized to weight. P7 *Smn^+/+^* vs SMNΔ7 HC+HX ** *P*=0.0061. P7 *Smn^+/+^* N to HC+HX ** *P*=0.0061. P14 *Smn^+/+^* vs SMNΔ7 N *** *P*=0.0002. P14 *Smn^+/+^* N to HC * *P*=0.0321, N to HC+HX * *P*=0.0112. (**B**) Minute ventilation (mL/min/gram) normalized to weight. P7 *Smn^+/+^* vs SMNΔ7 HC+HX * *P*=0.0438. P7 *Smn^+/+^* N to HC+HX *** *P*=0.0006, HC to HC+HX * *P*=0.0177. P14 *Smn^+/+^* vs SMNΔ7 N ** *P*=0.0078. P14 *Smn^+/+^* N to HC * *P*=0.0138, N to HC+HX ** *P*=0.0039. (**C**) Inspiratory time measured in seconds. P14 *Smn^+/+^* vs SMNΔ7 HC * *P*=0.0453, HC+HX *** *P*=0.0008. (**D**) Expiratory time measured in seconds. P14 *Smn^+/+^* vs SMNΔ7 N * *P*=0.0210. P14 SMNΔ7 N to HC * *P*=0.0256. P7 n=10 for each group. P14 *Smn^+/+^* n=12, SMNΔ7 n=8. mL = milliliter, sec = second min = minute.


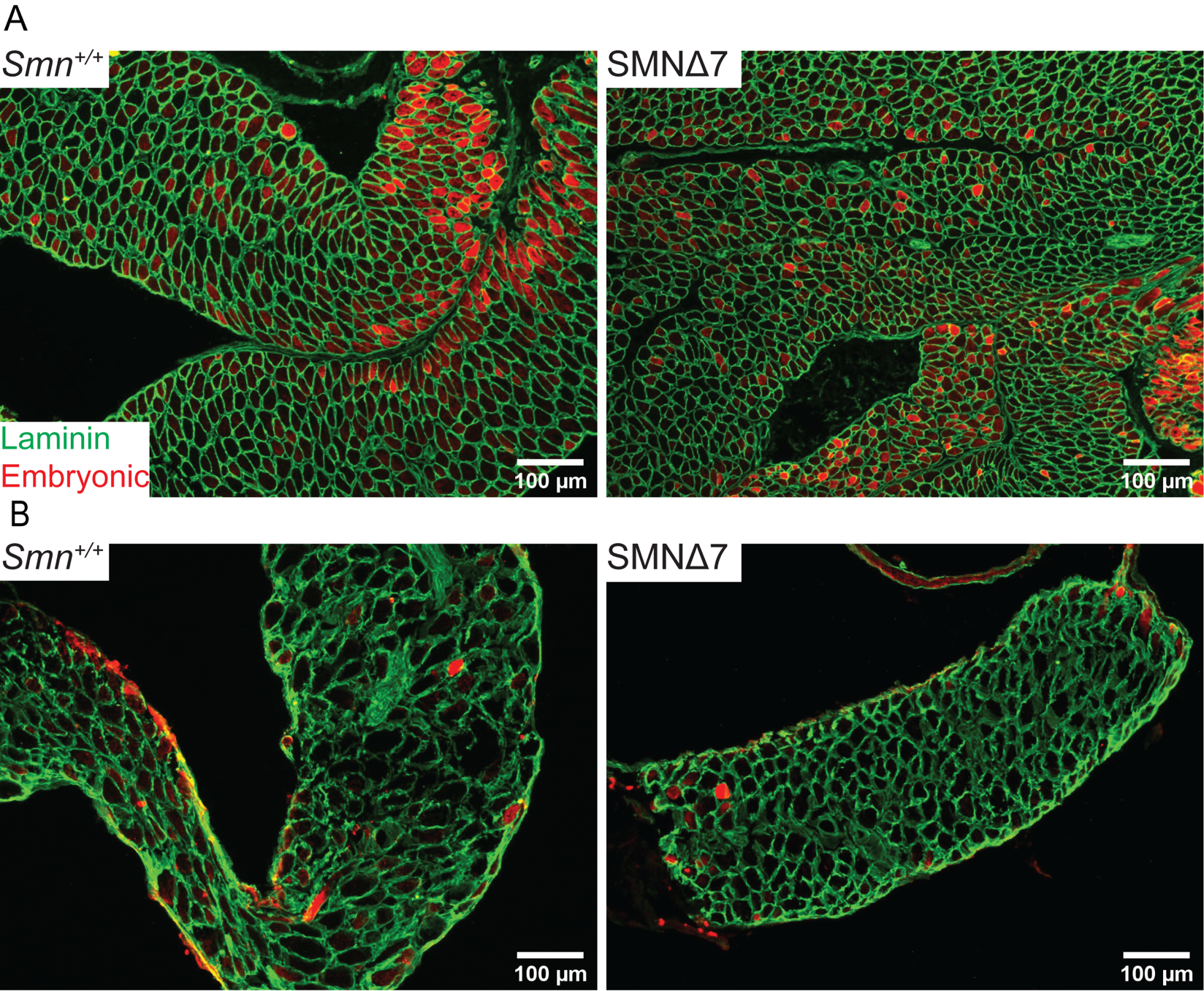


**Supplemental 2** Representative images of diaphragm muscle fiber types. (**A**) P7 diaphragms with laminin (green) and embryonic fibers (red) represented. (**B**) P14 diaphragms with laminin (green) and embryonic fibers (red) represented. All representative images are taken at a 10X magnification.


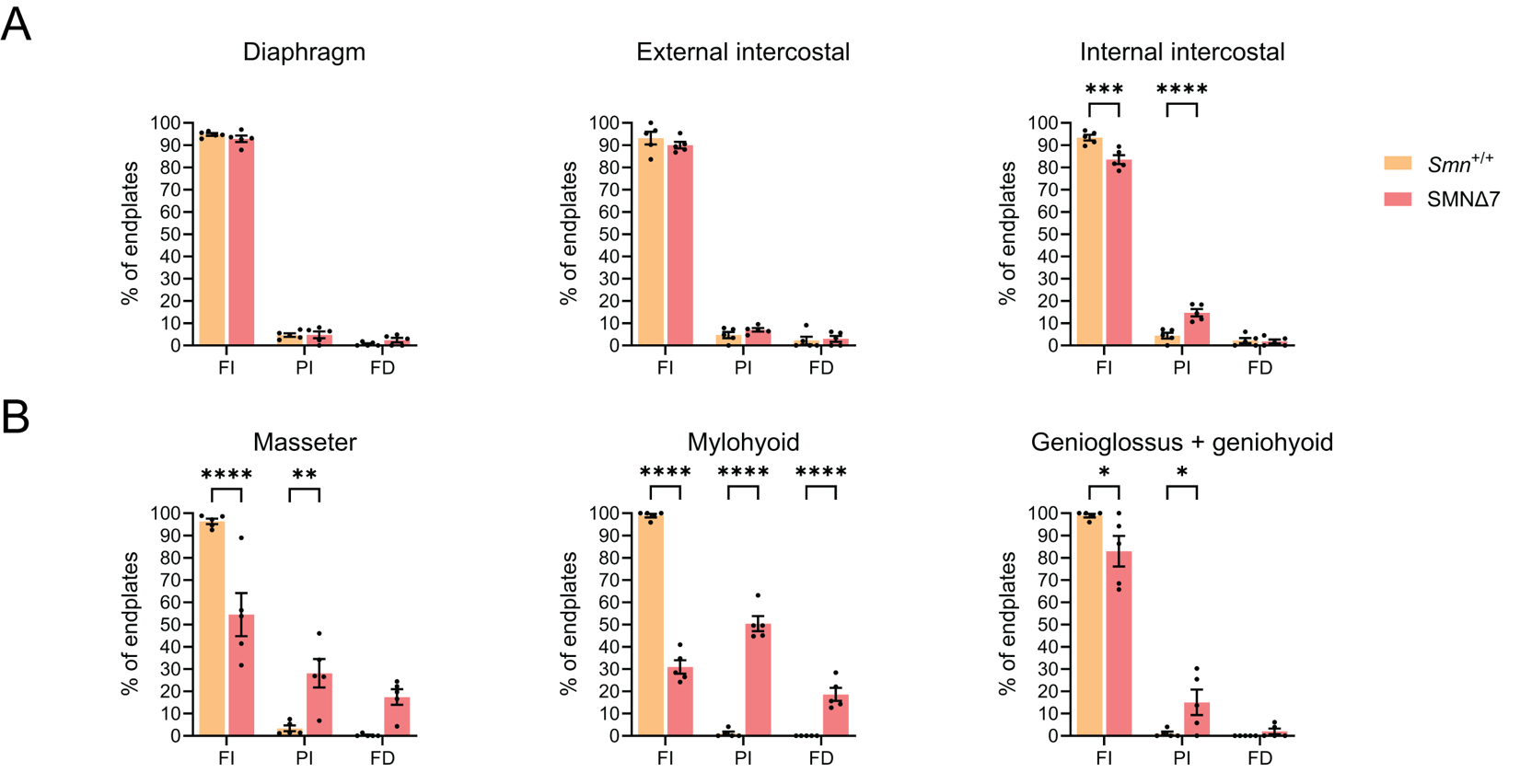


**Supplemental 3** SMNΔ7 NMJs showed denervation at P14. *Smn^+/+^* mice are represented as gold bars and SMNΔ7 mice are represented as peach bars. (**A,B**) NMJ quantification as a percentage of analyzed endplates. FI=fully innervated, PI=partially innervated, FD=fully denervated. (**A**) P14 lower respiratory tract. Internal intercostal FI *** *P*=0.0002, PI **** *P*=<0.0001. (**B**) Oral cavity & upper respiratory tract muscles at P14. Masseter FI and PI **** *P*=<0.0001. Mylohyoid FI, PI, and FD **** *P*=<0.0001. Genioglossus+geniohyoid FI * *P*=0.0163, PI * *P*=0.0399. N = ³ 4 for all groups.


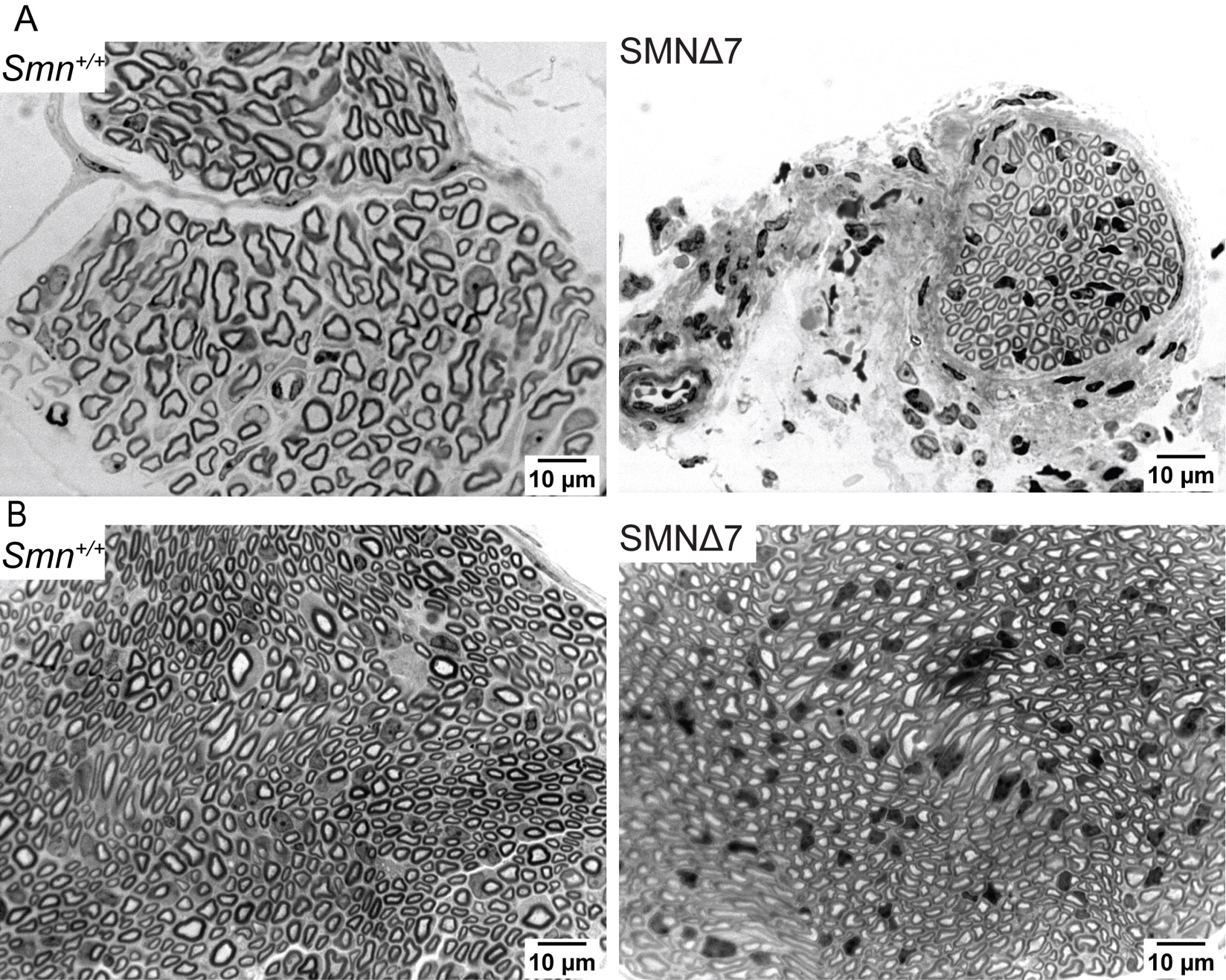


**Supplemental 4** Representative images of P14 phrenic and hypoglossal nerves. (**A**) P14 phrenic nerve cross sections stained in toluidine blue. (**B**) P14 hypoglossal nerve cross sections staining in toluidine blue. All images were taken at 100X.
